# A simple novel technique for the management of a dentoalveolar fracture in a pediatric patient using a vacuum-formed splint

**DOI:** 10.34172/joddd.2020.010

**Published:** 2020

**Authors:** Kumar Nilesh, Ashish Mahamuni, Swapnil Taur, Aaditee V. Vande

**Affiliations:** ^1^Department of Oral & Maxillofacial Surgery, School of Dental Sciences, KIMSDU, Karad, Maharashtra, India; ^2^Department of Pedodontics & Preventive Dentistry, School of Dental Sciences, KIMSDU, Karad, Maharashtra, India; ^3^Department of Prosthodontics, School of Dental Sciences, KIMSDU, Karad, Maharashtra, India

**Keywords:** Fracture, mandible, paediatric, thermoformed splint, trauma

## Abstract

This paper reports a novel, minimally invasive, simple technique for the treatment of a displaced dentoalveolar fracture using a vacuum-formed splint in a 12-year-old pediatric patient. Vacuum formed splints have been reliable treatment options with limited morbidity and discomfort compared to other traditionally used procedures.

## Introduction

Traumatic injuries to the dentofacial structures in adolescents due to reasons such as falls, sports injury, or road traffic accidents might often lead to the avulsion of anterior teeth, dentoalveolar, and jaw fractures. The incidence of dentoalveolar injury in children is variable, commonly involving the anterior maxillary segment followed by the anterior mandible.

Decision-making in the management of pediatric fractures is more challenging as compared to adults due to factors such as smaller jaw size, centers of active bone growth, and unerupted permanent teeth. The goal in treating pediatric fractures is to restore the underlying bony structure to its pre-injury position as early as possible. The treatment of choice should be ^minimally^ invasive and result in the restoration of occlusion with minimal residual esthetic and functional impairment. The management of dentoalveolar fractures partly relates to the treatment of jaw fracture and partly to that of tooth luxation. As a general rule, conservative therapy with long-term follow-up should be favored when treating facial trauma in adolescents, especially when it involves the tooth-bearing regions. Currently, the various methods of stabilizing displaced dentoalveolar fractures include the application of arch bars and metal (cap) splint or acrylic plates fixed using circum-mandibular wiring under general anaesthesia.^1^

Vacuum-formed splints are traditionally used as night guards to prevent occlusal wear in chronic bruxers and temporomandibular joint disorder patients. Lloyd^2^ in 2001 first reported the use of vacuum-formed splint for the treatment of mandibular fractures. The splint is made up of a transparent thermoplastic sheet with the help of a vacuum-forming machine, which is then adapted to the individual dentition. The splint obtains dentoalveolar support through close adaptation to the teeth and alveolus. The splint attains exact anatomical duplication of the patient’s dentition, thus providing excellent three-dimensional stability at the fracture site. The vacuum-formed splint might be a useful alternative to arch bars in managing dentoalveolar fractures, especially in pediatric patients with primary or mixed dentition, where arch bar adaptation is difficult due to retention problems. This technique is minimally invasive and can be carried out in outpatient settings, thus avoiding hospitalization and general anesthesia procedures. This paper reports the use of a vacuum-formed splint in the management of a dentoalveolar fracture of the anterior mandibular segment in a young male patient. The paper also reviews the literature for the use of this new technique in managing jaw fractures in pediatric patients.

## Case report

**Figure 1:**
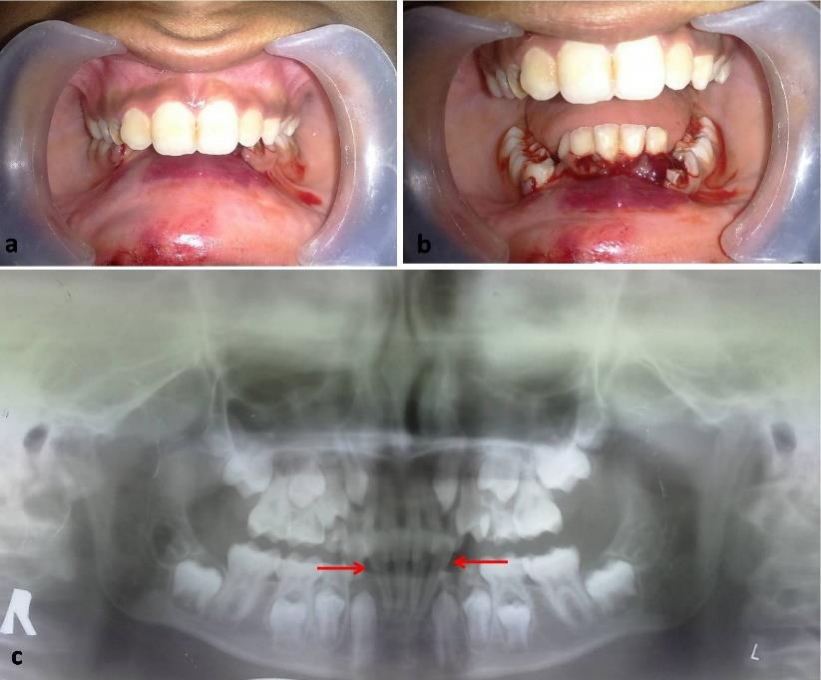
Clinical presentation showing deep bite (a), and displaced mandibular anterior dentoalveolar segment associated with labial echymosis (b). Panoramic radiograph showing the fracture segment (c).

A 12-year-old boy was referred to the oral and maxillofacial surgery clinic with a chief complaint of bleeding from the mouth as a result of a fall from a bicycle. The patient reported on the same day of the injury and received immediate attention. The child was conscious and cooperative at the time of examination with vital signs within the normal limits. There was no history of loss of consciousness, bleeding from the nose and ears, vomiting, or convulsions. On the extraoral examination, the patient presented a normal (straight) facial profile with swelling over the lower lip and chin. Intraoral examination showed labial ecchymosis in the mandibular anterior region and a step deformity between the distal aspect of #32 and the distal aspect of #83. The anterior dentoalveolar segment was displaced superiorly and lingually, causing a deep bite (Figure 1a and 1b). The panoramic radiograph revealed a superiorly displaced dentoalveolar segment of the anterior mandible, confirming a diagnosis of dentoalveolar fracture (Figure 1c).

The clinical findings and diagnosis of mandibular dentoalveolar fracture were explained to the patient’s parents. Various treatment modalities, including arch bar fixation, cap splint, acrylic splint with circum-mandibular wiring, and immobilization with vacuum-formed splints, were discussed. The option of using vacuum-formed splints applied in an outpatient setting was chosen and executed under local anesthesia.

**Figure 2:**
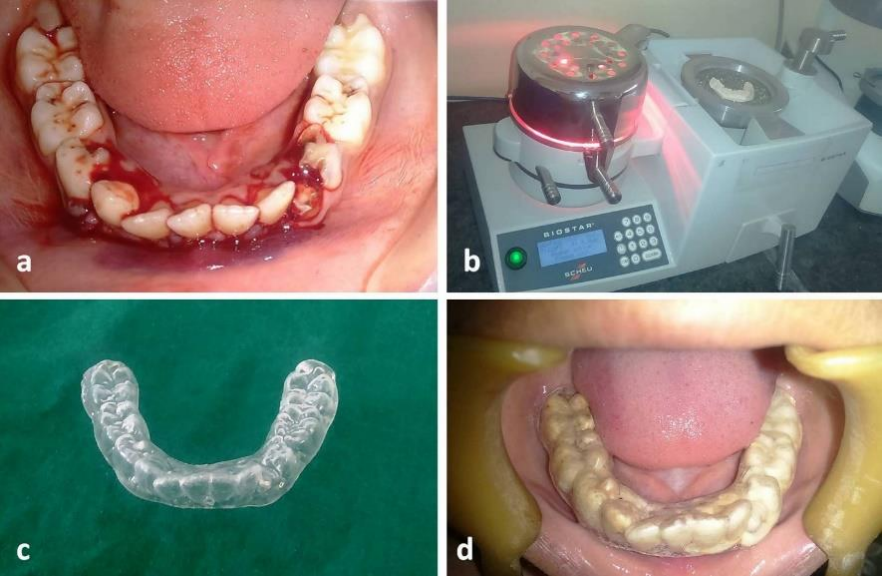
The reduction of the fractured segment (a), pressure molding machine (b), the prepared vacuum-formed splint (c), and splint-cemented to the lower dentition (d).

**Figure 3:**
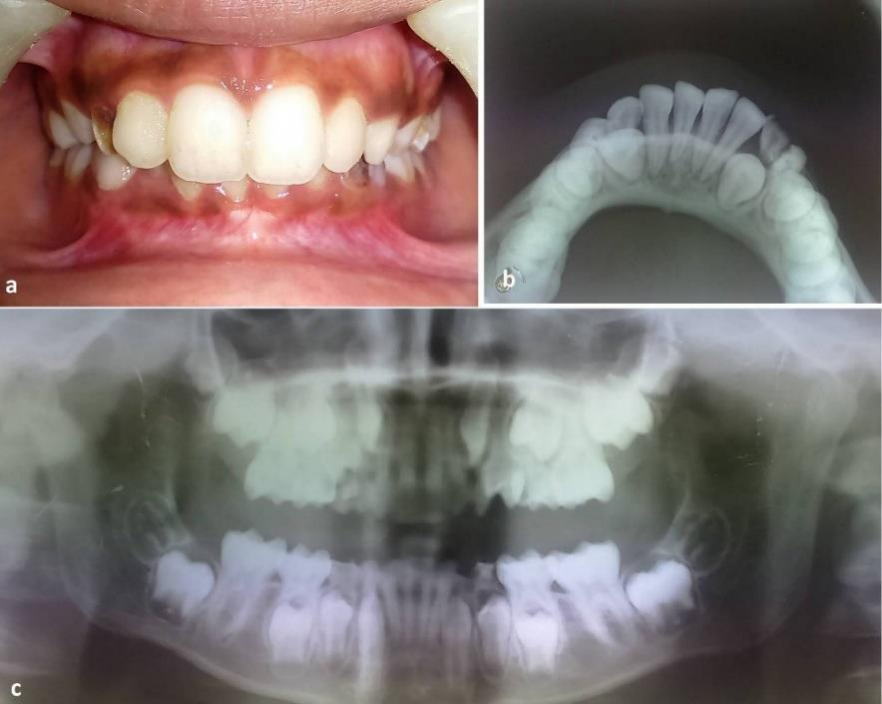
Postoperative occlusion after the removal of the splint (a). Mandibular occlusal radiograph (b) and panoramic radiograph (c) showing bone healing at the fracture site.

Under bilateral inferior alveolar nerve block, the displaced dentoalveolar segment was repositioned with finger pressure (Figure 2a). Bilateral occlusion was evaluated visually and with the help of articulating paper. Once adequate occlusion was attained, a mandibular impression was taken using alginate impression material, and the dental cast was obtained. A 1-mm transparent thermoplastic foil (Scheu Duran, Liberal Traders Pvt. Ltd., New Delhi, India) was pressed onto the mandibular cast with the help of a Biostar pressure molding machine (Schue Dental, GmbH, 58642, Iserlohn, Germany; temperature: 220ºC and pressure: 4.8 bars for 30 seconds) (Figure 2b) for the fabrication of a vacuum-formed splint. The splint margins were trimmed using scissors to engage the undercut area 2 mm below the free marginal gingiva for additional mechanical retention (Figure 2c). Overextension of the splint was avoided for the maintenance of oral hygiene. The splint was cemented onto the mandibular dentition using type I glass-ionomer cement (Figure 2d). The splint was maintained for three weeks. Prophylactic antibiotic (cefixime, 200 mg, twice a day) and analgesic (a combination of paracetamol, 325 mg, and ibuprofen, 200 mg, twice a day) were prescribed for a week. Postoperative instructions of soft diet and use of mouthwash were explained to the parents. Postoperative monitoring was undertaken on a weekly basis. The splint was removed after three weeks. A stable occlusion was attained at the end of three weeks (Figure 3a). Panoramic and mandibular occlusal radiographs taken at one-month follow-up showed radiographic evidence of healing (Figure 3b,c).

## Discussion

Dentoalveolar fractures are injuries affecting teeth with its supporting alveolar bone. It commonly presents as a displaced dentoalveolar segment, tooth mobility, occlusal disturbance, and hematoma into the adjacent oral mucosa. The management of a dentoalveolar fracture involves the reduction of the displaced dentoalveolar segment and its stabilization for 2‒4 weeks. Various stabilization techniques have been reported in the literature, including arch bar, metal cap splints, acrylic plates fixed with per-alveolar or circum-mandibular wiring, dental splinting (using wire-composite, fiber-glass, porcelain veneer), interdental ligature wiring, bone screw-plate fixation, and suturing.^1^ Methods of immobilization by dental splinting and ligature wiring have been effective in treating luxated/avulsed teeth and minimally displaced dentoalveolar segments. Traditionally the two most commonly used modalities for stabilization of displaced dentoalveolar fracture include the application of arch bars fixed with inter-dental wiring and use of metal (cap) splint/acrylic plates fixed using circum-mandibular wiring.^2^ In pediatric patients with mixed dentition, the height of the contour of deciduous teeth is below the gingival level. Thus, the application of arch bars is not feasible. Arch bars also provide lower stability due to root resorption of deciduous teeth and incomplete formation of the roots of permanent teeth. However, splinting with an acrylic plate requires circum-mandibular/peri-alveolar wiring, necessitating hospitalization to carry out the procedure under general anesthesia.

The use of a vacuum-formed splint cemented to the dentition is a novel and relatively simple method of stabilization of dentoalveolar fractures. Conventionally, these splints are used as occlusal night guards in patients with bruxism. The use of a vacuum-formed splint for the treatment of jaw fracture was first reported by Lloyd et al^2^ in 2001 for the management of condylar fracture in a child patient.A review of the English literature for the use of vacuum-formed splints in the treatment of jaw fractures in pediatric patients revealed six reported cases (Table 1).^2-7^ It has been used in both the male and female patients with an age range of 4‒13 years. All the reported cases were related to the management of mandibular fractures, including three parasymphysis, one symphysis, and one condyle fracture, and one multiple mandibular fractures. In the present case, the splinting technique was successfully used in the management of a displaced dentoalveolar fracture of the anterior mandible.

**Table 1:** A review of vacuum-formed splints in the treatment of pediatric jaw fractures

**Sr. no.**	**Author; Year of reporting**	**Age/** **Sex**	**Site of fracture**	**Stabilization method**	**Modification**	**Outcome**
**1**	**Lloyd T. et al; 2001** ^2^	13/F	Left condyle fracture	Cementation	Incorporated arch bar hooks for IMF	Adequate mouth opening and occlusion in centric relation
**2**	**Emmanuel A. et al; 2010** ^3^	4/F	Parasymphysis fracture	Circum-mandibular wiring	None	Stable occlusion
**3**	**Choubey S. et al; 2014** ^4^	9/M	Parasymphysis fracture	Cementation	None	No signs of inflammation and healthy healing at fracture site
**4**	**Reddy KH.et al; 2016** ^5^	4/M	Parasymphysis fracture	Circum-mandibular wiring	None	No mobility at the fracture site
**5**	**Nilesh K. et al; 2016** ^6^	5/M	left parasymphysis, left ramus and right body of mandible	Cementation	Incorporated arch bar hooks for IMF	Good dento-gingival health and stable occlusion
**6**	**Sanu O. et al; 2017** ^7^	6/M	Symphysis fracture	Circum-mandibular wiring	None	Stable occlusion
F= female, M=male,						

Vacuum-formed splints are bio-acrylic splints which are useful alternatives to arch bars where the gap between the two adjacent teeth are wide, less anchorage is present due to periodontally compromised teeth, and when support is available only from deciduous teeth and immature permanent teeth (with incomplete root formation) in the mixed dentition period. They are inexpensive and easy to fabricate and are prepared by molding a transparent thermoplastic foil over the cast of the patient in a vacuum molding machine. The transparency of the material allows visualization of the underlying dentition for easy checking of the adaptation and occlusion.^3^ The splint is closely adapted over the occlusal surfaces of teeth, providing good interdigitation of dentition. The smooth surface makes it easier to use it without any risk of injury to periodontal tissues and improves the patient’s compliance in maintaining adequate oral hygiene, especially in younger age groups.^2,4^ The ease of cutting the splint with scissors makes the trimming of the overextended material easy and thereby shortens the overall fabrication and chairside time. Due to the even thickness of the splint, there are no premature contacts, and as the splint covers the entire occlusal surface, it avoids supra-eruption of the dentition. The flexibility of the splint allows passive adaptation over the dentoalveolar tissue, thus permitting an easy fit into undercuts and over teeth, thereby providing excellent retention. Its three-dimensional flexibility also allows physiologic tooth mobility without transferring orthodontic forces to the splinted teeth. On the other hand, the material also provides sufficient mechanical stiffness to withstand masticatory shearing forces.^5,6^

Vacuum-formed splints are advantageous as they are non-invasive and preserve the anatomy of the mandible and the developing tooth buds, permitting mastication and speech without any functional deformity. The closely contoured margins of the plastic splint allow mechanical retention by engaging the dentition and the bone undercuts. However, additional means of stabilization is required when treating a displaced fracture segment, which can be achieved mechanically by cirucum-mandibular wiring or chemically by using a luting cement to bond the splint with the teeth. In the present case, the retention of the splint was attained using glass-ionomer cement. The use of glass-ionomer cement has an additional benefit of fluoride release, thus minimizing the decalcification of the deciduous teeth in younger patients.^6^ The splint was maintained for three weeks. The patient did not report any discomfort during this period. The gingival health was good on the removal of the splint, with stable occlusion and alignment of the dentoalveolar segment.

## Conclusion

Selection of the splinting method for the immobilization of jaw fracture in pediatric patients must meet specific criteria, including ease of fabrication, application of only passive forces on the teeth, no injury to the soft tissues, underlying bone, and the developing tooth buds, maintain stable occlusion, allow maintenance of good oral hygiene, and easy removal. Vacuum-formed splints offer various advantages. They are an easy and efficient technique for the management of pediatric fractures, including dentoalveolar injuries, and need to be incorporated more often in routine clinical practice.

## Authors’ contributions

KN: drafting of the manuscript, conducting the clinical procedure, final approval of the manuscript; AM: participation in conducting the clinical procedure, preparation of the manuscript; ST: conducting the clinical procedure; AVV: Drafting of the manuscript, final approval of the manuscript. All authors have read and approved the final manuscript.

## Acknowledgments

None.

## Funding

None.

## Competing Interests

The authors declare that they have no competing interests.

## Ethics approval

Not applicable.
